# Effects of ambient humidity and surface topography on fingermark recovery from PLA 3D‐printed surfaces

**DOI:** 10.1111/1556-4029.70198

**Published:** 2025-10-20

**Authors:** Laura J. Vera Stimpson, Breeshea Robinson, Julie Bramble, Andrew Langley, Diana‐Madalina Suciu

**Affiliations:** ^1^ School of Business, Law and Policing Canterbury Christ Church University Kent UK; ^2^ School of Engineering, Technology and Design Canterbury Christ Church University Kent UK; ^3^ Division of Natural Sciences University of Kent Kent UK

**Keywords:** 3D printing, fingermarks, fused deposition modeling (FDM), fused filament fabrication (FFF), polylactic acid (PLA), surface orientation, surface texture

## Abstract

The increasing accessibility of 3D printing, made possible by the affordability of equipment and materials, has led to its widespread adoption in both domestic and industrial applications, with polylactic acid (PLA) being a commonly used material. The layer‐by‐layer deposition process in fused deposition modeling creates surface texture variations that significantly influence the development and recovery of latent fingermarks. This study examined the effect of raster lines on fingermark development by depositing latent fingermarks on the X, Y, and Z faces of 3D‐printed PLA objects. Powder development was applied both along and against the 3D print grain. Development against the grain caused excess powder accumulation within raster lines, partially obscuring ridge detail. In contrast, applying powder along the grain minimized accumulation, enabling clearer visualization of ridge features. Top and side surfaces generally yielded higher quality grades, attributed to smoother surfaces from better interlayer bonding. However, raster lines created discontinuities in ridge transfer, hindering coincident sequence determination. Cyanoacrylate ester fuming effectively addressed this limitation, producing continuous ridge detail on top and side surfaces, and leading to higher quality grades.

**Highlights**

- 3D print orientation alters texture, impacting fingermark enhancement on FDM surfaces.
- Fingermark quality on PLA 3D prints degrades quickly over short time periods.
- Layer lines in FDM 3D prints affect fingermark deposition, causing voids.
- Fingermark recovery methods assessed include powders and CEF techniques.
- Findings highlight difficulties in fingermark recovery from 3D‐printed items.

## 1 INTRODUCTION

Three‐dimensional (3D) printing, also known as additive manufacturing, has emerged as a transformative technology since its inception [1] in 1969 and has advanced rapidly in recent years, expanding its accuracy and reliability within various fields, including manufacturing [2], medicine [3], aerospace [4], and defense [5]. Initially developed for prototyping purposes, 3D printing has undergone significant evolution in the spectrum of materials used, ranging from polymers [6] and metals [7] to ceramics [8] and biological tissues [9]. Technological advancements in 3D printing, including stereolithography, fused deposition modeling (FDM), also known as fused filament fabrication, selective laser sintering, and multimaterial printing, have facilitated the creation of objects with functional properties.

The relationship between these processes lies in the ability of the material to solidify or locally fuse, enabling the formation of the desired structure in a layer‐by‐layer manner. Among these techniques, FDM is the most widely utilized [10]. It falls under the material extrusion category and employs thermoplastic materials as its primary raw component. FDM uses a build plate, nozzle, and filament spool. The process begins by creating a 3D model of the object in Standard Tessellation Language (STL) format using geometry creation software. This model is imported into slicing software, which divides it into thin 2D layers, generating a path for the nozzle's movement, controlled by a 3‐axis system. The nozzle moves in the X‐Y plane to deposit the first layer, and after each layer is completed, the nozzle moves upward in the Z‐direction by a set layer thickness, fusing the new layer with the previous layer.

FDM is a widely used 3D printing technology due to its cost‐effectiveness, accessibility, and versatility. Its straightforward mechanism allows for low production costs and affordable raw materials, making it suitable for various purposes from hobbyist projects to industrial applications. This has led to its widespread adoption [11] with growth predicted to rise exponentially from 2024 to 2030, highlighting the increasing significance of the technology [12].

Polylactic acid (PLA) is the most widely used material in FDM printing due to its biodegradability and environmentally friendly nature. As the most important thermoplastic in the field [13], PLA offers several advantages, including being recyclable and easily processed through thermal methods. Its relatively low melting point (150–160°C) reduces energy consumption during printing, making it an efficient choice. Additionally, in the FDM process, the mechanical strength and surface quality of printed objects can be optimized by adjusting polymer properties and printing parameters such as nozzle temperature, layer thickness, and raster orientation. With an annual growth rate [14] of 20%, PLA continues to be a dominant material in 3D printing, combining sustainability with versatility.

The increasing availability of 3D‐printing equipment, coupled with the widespread accessibility of computer‐aided design files, has facilitated the production of objects, enabling individuals with rudimentary knowledge to produce a diverse array of items, including regulated objects such as knives [15] and firearms [16]. Consequently, the forensic community is poised to witness an upsurge in crime scene exhibits manufactured through 3D printing methods. It is imperative to develop suitable methods for the visualization, enhancement, and recovery of evidence from these objects.

Among potential evidentiary considerations is fingermark evidence, where comparison of a fingermark left at a crime scene with a set of fingerprints taken under controlled conditions is a cornerstone of forensic science [17, 18]. This is due to the practical uniqueness of fingermarks, allowing for individualization, as well as their widespread acceptance within the criminal justice system. However, the reliability of fingermark evidence is dependent upon the enhancement, recovery, preservation, and subsequent analysis methods. The substrate upon which fingermarks are deposited, along with the chosen development technique, significantly influences the quality of the developed fingermarks [19]. While the fingermark visualization manual [20] offers guidance on suitable development methods for recovering fingermarks from various surfaces, there is a notable absence of guidance on recovering fingermarks specifically from 3D‐printed objects. This study aims to investigate the impact of three distinct surface textures commonly found in 3D‐printed objects on the deposition and recovery of fingermarks. By using PLA as the printing material, this research seeks to evaluate how surface texture influences fingermark visibility and recovery effectiveness, providing insights into forensic applications involving 3D‐printed surfaces.

## 2 MATERIALS AND METHODS

### 2.1 3D printing setup and materials

An ELEGOO Neptune 4 filament printer, with a build volume of 225 × 225 × 265 mm^3^ and a 0.4 mm nozzle diameter, was used to produce all models. The samples, each in the shape of a cube measuring 2.54 cm per side, were printed using ELEGOO PLA+ filament (1.75 mm diameter) in either white or black. A textured polyetherimide (PEI) flexible build plate was used for all models. G‐code generation was obtained using UltimakerCura 5.9 slicing software [21], with Table 1 detailing the specific printer parameters used.

**TABLE 1:** Parameters used for FDM printing.

Printing parameter	Setting
Infill pattern	Grid
Infill density	15%
Infill speed	250 mm/s
Nozzle temperature	200°C
Bed temperature	60°C
Number of layers	125
Layer height	0.2 mm
Raster angle	45°
Raster pattern	Contour‐raster

### 2.2 Deposition and aging of fingermarks

Prior to fingermark deposition, the cubes were dried in an oven at 50°C for 2 h. Following drying, the cubes were transferred to a sealed desiccator and allowed to cool to room temperature. A single female donor provided all fingermark samples for this study. The deposition process was controlled to maintain a uniform force and angle, set to 1.5 N and 90°, respectively.

Latent fingermarks were aged under two distinct environmental conditions for 1, 3, 5, and 7 days. In this study, the 1‐day aging period refers to a deposit‐recovery method in which fingermarks were deposited, allowed to dry under the specified environmental conditions, and subsequently enhanced and recovered. This period represents the initial state of the fingermarks and serves as a baseline for comparison with extended aging.

- *Environment 1* represented a standard laboratory setting, where the temperature fluctuated between 19°C and 23°C, with an average temperature of 22°C and an average of 27% relative humidity.
- *Environment 2* consisted of a vacuum desiccator, where the temperature varied between 19°C and 23°C, with an average of 22°C. However, the relative humidity in this environment was maintained at approximately 5%.

### 2.3 Enhancement and recovery of latent fingermarks

Latent fingermarks were enhanced using black powder (Tetra) and black magnetic powder (Tetra). Black powder was applied using a fine animal hair brush, while magnetic powder was applied using a magnetic wand. The application procedure adhered to the standardized procedures outlined in the Fingermark Visualization Manual [20]. Following enhancement, white gel lifts (SceneSafe) were used to recover the developed fingermarks, facilitating subsequent analysis.

Cyanoacrylate ester fuming (CEF), also known as cyanoacrylate fuming, was used for the chemical development of deposited fingermarks. A 0.07 m^3^ fuming chamber was constructed using vertically stacked plastic storage boxes, designed to accommodate all necessary components. Within the enclosure, a hot plate, a beaker of hot water, and aluminum tins containing five drops (~0.18 g) of cyanoacrylate‐based superglue were used to facilitate the fuming process. During development, exhibits were suspended above the hot plate. To maintain controlled conditions, all seams of the chamber were sealed prior to initiating the fuming process. The temperature inside the enclosure was gradually increased to 120°C and sustained for 90 minutes to allow for effective polymerization of the cyanoacrylate on the fingermark ridges.

### 2.4 Assessment of developed fingermarks

The quality of the developed fingermarks was evaluated using a grading system chosen in consultation with a fingerprint expert. To ensure an objective assessment, the classification method established by Castelló [22] (Table 2) was utilized alongside expert analysis to categorize the clarity, detail, and overall distinctiveness of the recovered fingermarks.

**TABLE 2:** Fingermark grading scale [22].

Grade	Description
0	No visible print
1	Poor quality, very few visible ridges
2	Poor quality, some ridge detail visible or partial mark with limited characteristics
3	Reasonable quality, ridge detail, and some characteristics visible, identification possible
4	Good quality print, ridge detail, and characteristics visible, probable identification
5	Excellent quality, full mark, very clear, and identification assured

The fingermarks were examined visually to assess their quality and characteristics. This included evaluating their clarity, ridge detail, and overall suitability for further analysis. Magnification was achieved through a linen tester at 10x magnification.

### 2.5 Surface characteristics

The surface characteristics of the 3D‐printed exhibits were analyzed using scanning electron microscopy (SEM). SEM analysis was performed using a Hitachi TM4000plus SEM instrument, and electron images were acquired using the proprietary Hitachi SEM control software. All images were collected under vacuum conditions, with an electron accelerating voltage of 5 kV.

## 3 RESULTS AND DISCUSSION

### 3.1 Surface characterization

The surface texture of FDM prints varies across different faces of a 3D‐printed object due to the layer‐by‐layer deposition process, where the nozzle extrudes thermoplastic material following predefined intra‐layer filling patterns. There are three primary intra‐layer filling strategies: raster (zigzag), contour, and a contour‐raster combination [23]. The contour‐raster combination is widely adopted due to its balance between filling efficiency, surface quality, and build time. This hybrid approach is commonly used as the default setting in slicing software such as UltiMaker Cura [21]. In the contour‐raster combination, the extruded thermoplastic is generally deposited along the X‐Y axis at raster angles of ±45° between adjacent layers. To enhance interlayer bonding and structural integrity, the deposition tracks of successive layers are interlaced, ensuring that extrusion lines do not align perfectly between layers. This interlacing technique improves mechanical strength, reduces delamination, and enhances surface quality by distributing stress more evenly across the printed surface [23].

**FIGURE 1:**
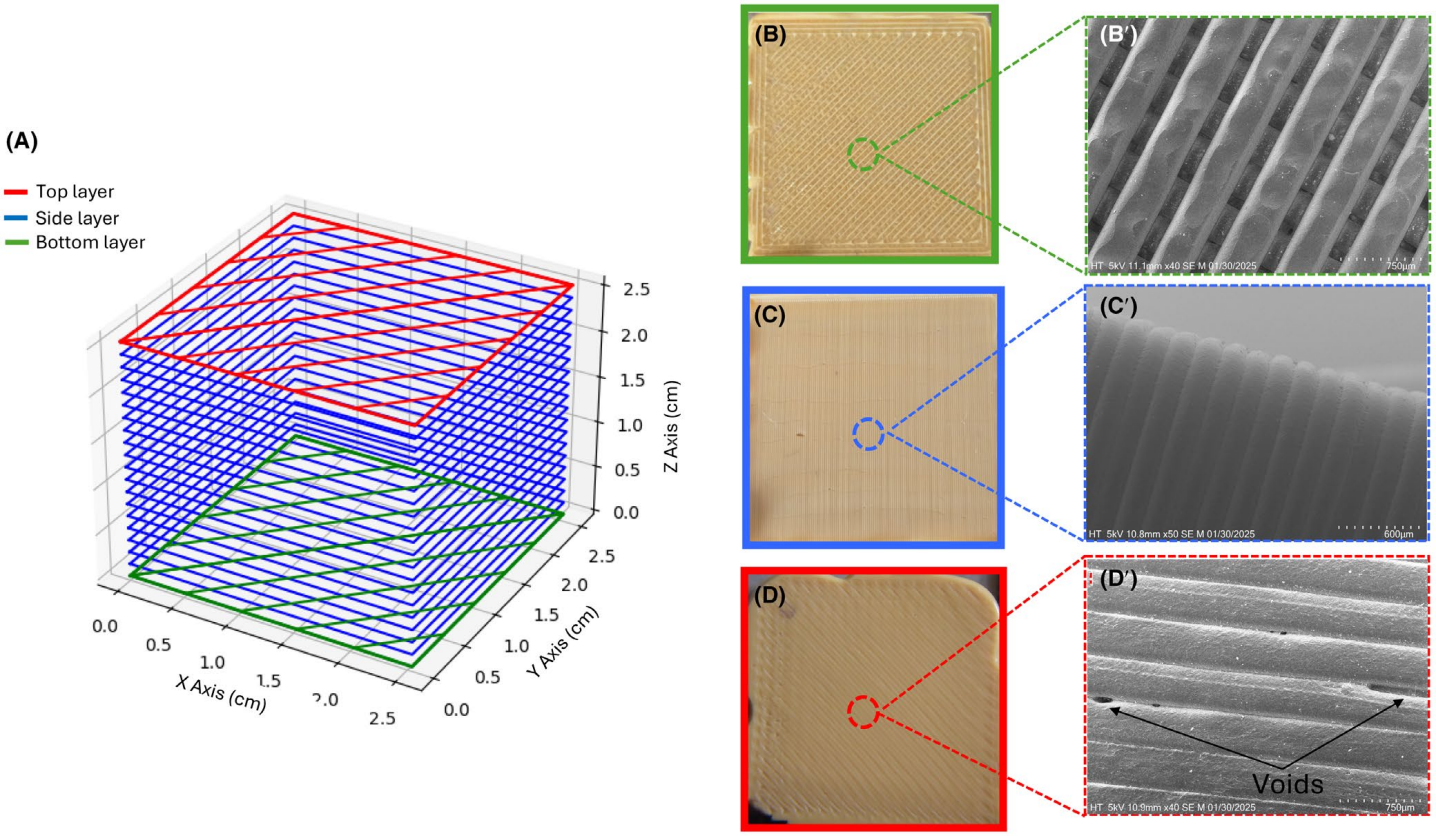
(A) 3D graphical representation of a 3D‐printed cube illustrating three distinct surface layers. (B–D) Provil® novo casts of each surface of the cube. (B′–D′) Scanning electron microscopy (SEM) images of the corresponding surfaces of the 3D‐printed cube.

The surface characteristics of each face of a 3D‐printed object are inherently determined by its orientation during printing (Figure 1A). The first layer (bottom layer) of a 3D‐printed object adheres directly to the build plate, anchoring the print and ensuring stability throughout the process. This adhesion is crucial for preventing detachment and maintaining print accuracy. When printed onto a conventional textured PEI build plate, the raster patterns maintain a semi‐cylindrical shape rather than fully merging with adjacent raster lines (Figure 1B). This is due to an interplay between surface texture, surface tension, and thermal dynamics. The textured surface of the build plate provides microscopic anchoring points restricting the lateral spread of the molten polymer. Additionally, PLA has a relatively high surface tension, which aids with the extruded filament maintaining its deposited shape (Figure 1B′).

The 3D‐printed objects gain height by stacking layers sequentially in the Z‐direction where the newly deposited layer adheres to the previous layer by a combination of thermal fusion and polymer chain entanglement. This creates a layered surface topology, where distinct horizontal striations are visible along the vertical surfaces (Figure 1C,C′).

The top layer, formed in the X‐Y plane, follows a similar raster pattern to the bottom layer but lacks direct adhesion to the build plate, resulting in a different surface finish (Figure 1D). The extruded heated thermoplastic bonds to the previously printed raster by partially melting the adjacent material and entangling the polymer chains. While this bonding process is essential for layer cohesion, it can lead to issues such as the formation of voids (Figure 1D′). These gaps may arise from the geometry of the rasters and layers, where inefficient packing results in unfilled spaces, or from temperature discrepancies that prevent full layer adhesion, thereby creating voids within the print.

### 3.2 Directionality implications

**FIGURE 2:**
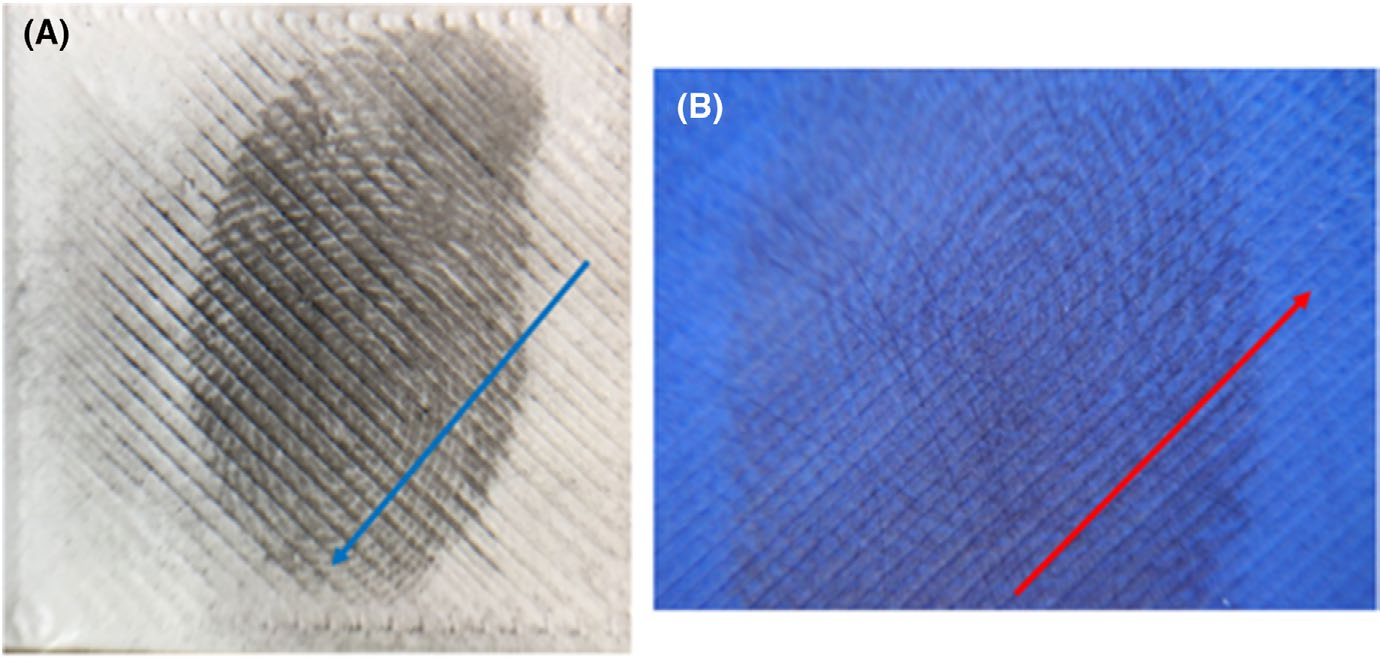
Effects of powder developer applied (a) against the grain and (b) along the grain. Arrows indicate direction of application.

The quality of developed fingermarks varied significantly depending on the direction in which the powder developer was applied, relative to the grain of the surface (Figure 2). Applying powder developer against the grain of the 3D‐printed surface (Figure 2A) resulted in an accumulation of excess powder within the raster lines, partially obscuring ridge details. Application of powder developer along the grain (Figure 2B) resulted in minimal accumulation of powder, allowing for clearer visualization of fingermark features.

### 3.3 Developer application deposit–recover

The application of powder developers along the grain on the three different surfaces of the 3D‐printed object allowed for the capture of fingermark detail; however, the quality of development varied depending on the powder type and surface characteristics.

**FIGURE 3:**
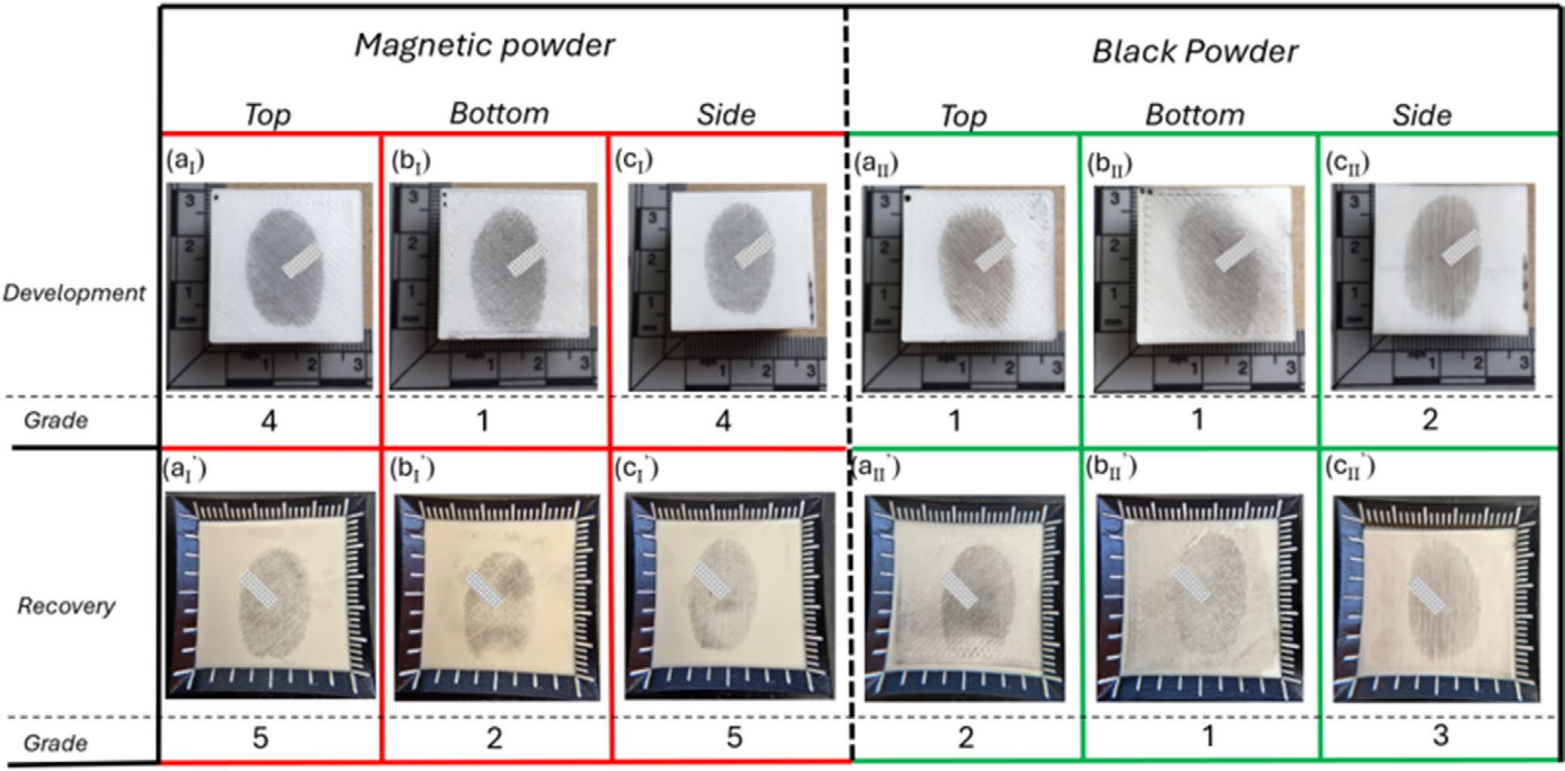
Development and recovery of latent fingermarks from (A) top, (B) bottom and (C) side surfaces of a 3D‐printed cube using (I) magnetic and (II) black powder.

Non‐magnetic granular powders (Figure 3) initially resulted in a lower development quality due to some powder accumulating within the raster lines of the 3D‐printed substrate. In contrast, magnetic powders, coated with ferromagnetic particles, reduced this issue by minimizing excess powder deposition and facilitating excess powder removal, leading to more effective fingermark development. However, after recovery with a gel lift, the grading of non‐magnetic powders showed improvement. The gel lift does not fully penetrate the raster lines of the surface, selectively capturing ridge detail on the surface while leaving excess powder trapped within raster lines. This selective lifting reduces background interference, enhancing the clarity of the recovered fingermark.

Ridge detail on the bottom surfaces of 3D‐printed objects showed the lowest fingermark quality due to the way the first layer of thermoplastic material is deposited onto the build plate during printing. The textured surface of the build plate affects how the material settles, creating a rough surface. This roughness is further increased by the raster pattern formed during printing, which consists of parallel lines of extruded plastic (Figure 1B′). Additionally, because the rough texture prevents the thermoplastic from fully merging, the lines of material do not bond completely, leaving small gaps between them. The uneven surface also causes the fingermark residue to spread inconsistently, further reducing its visibility.

The grades assigned to the top and side surfaces of 3D‐printed objects showed high associated grades, indicating their suitability for comparison purposes. Ridge detail was observable, due to smoother surfaces where effective bonding of adjacent layers was present. However, fingermark ridge detail transfer was influenced the layer lines characteristic of the 3D printing process. These led to incomplete ridge impressions being recovered, as fingermarks were deposited only on surface raster lines, resulting in gaps within the ridge detail of the fingermark. Such voids were evident both in direct visual examination of the object and in the recovered fingermark. This discontinuity in ridge detail causes challenges for coincident sequence determination, as the absence of complete ridge paths may hinder the alignment of minutiae points during fingermark comparison. Consequently, this can reduce the reliability of identification, increase the likelihood of erroneous exclusions, and complicate the forensic interpretation of fingermarks deposited on 3D‐printed surfaces.

**FIGURE 4:**
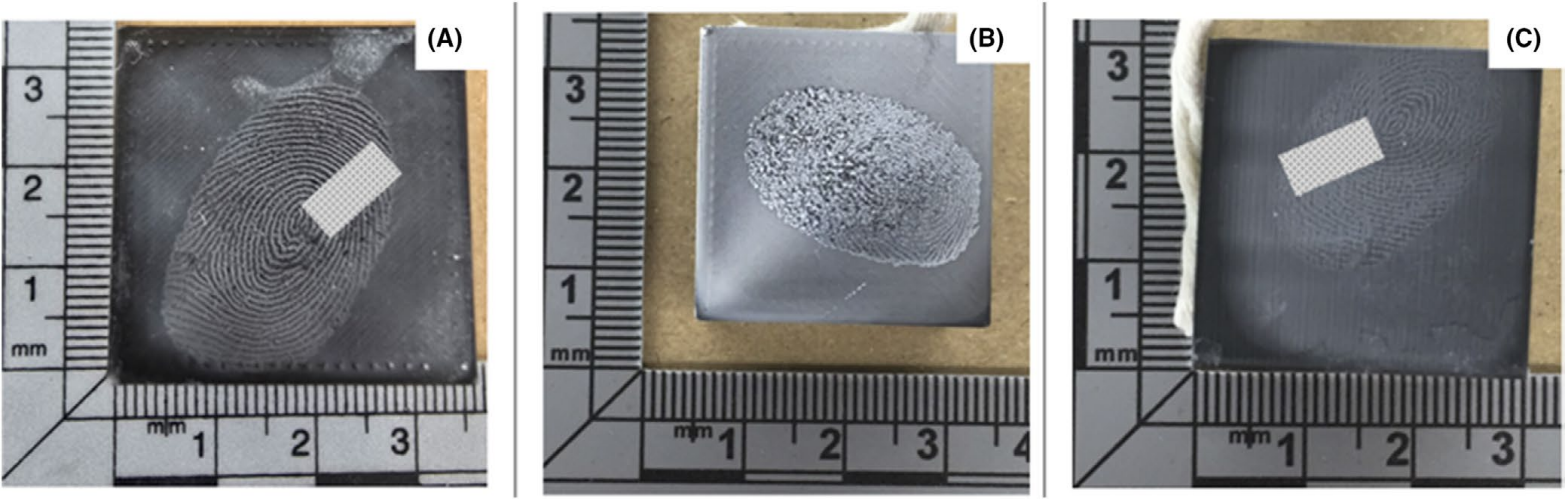
Latent fingermarks developed using CEF on the (A) top, (B) bottom, and (C) side surfaces of a 3D‐printed cube.

Issues with coincident sequence determination were overcome using CEF, as seen in Figure 4. Results obtained for the top and side surfaces showed complete ridge detail visualization, which resulted in high associated grades being assigned. CEF develops ridge detail through a polymerization reaction that results in a layer‐by‐layer accumulation of cyanoacrylate on the ridges of the deposited fingermark. This progressive accumulation builds up outward from the ridges, causing the development of fingermarks in a forward projection and preventing accumulation of material within surface depressions, as seen with powder‐based methods. Coincident sequence determination is possible as CEF develops uniformly across the surface, reducing the likelihood of incomplete ridge patterns. However, as shown in Figure 4B, fingermarks deposited on the bottom surfaces of 3D‐printed objects yielded an average grade of 1, indicating reduced development quality. While CEF is less affected by surface texture than powder‐based developers, the initial deposition quality of the latent fingermark remains a key factor. The surface texture, gaps, and porosity of the PLA material on the bottom surface disrupt the uniform transfer of ridge detail, leading to reduced clarity and completeness of friction ridge features, which may hinder forensic comparison and identification.

### 3.4 Development and recovery of aged latent fingermarks

**FIGURE 5:**
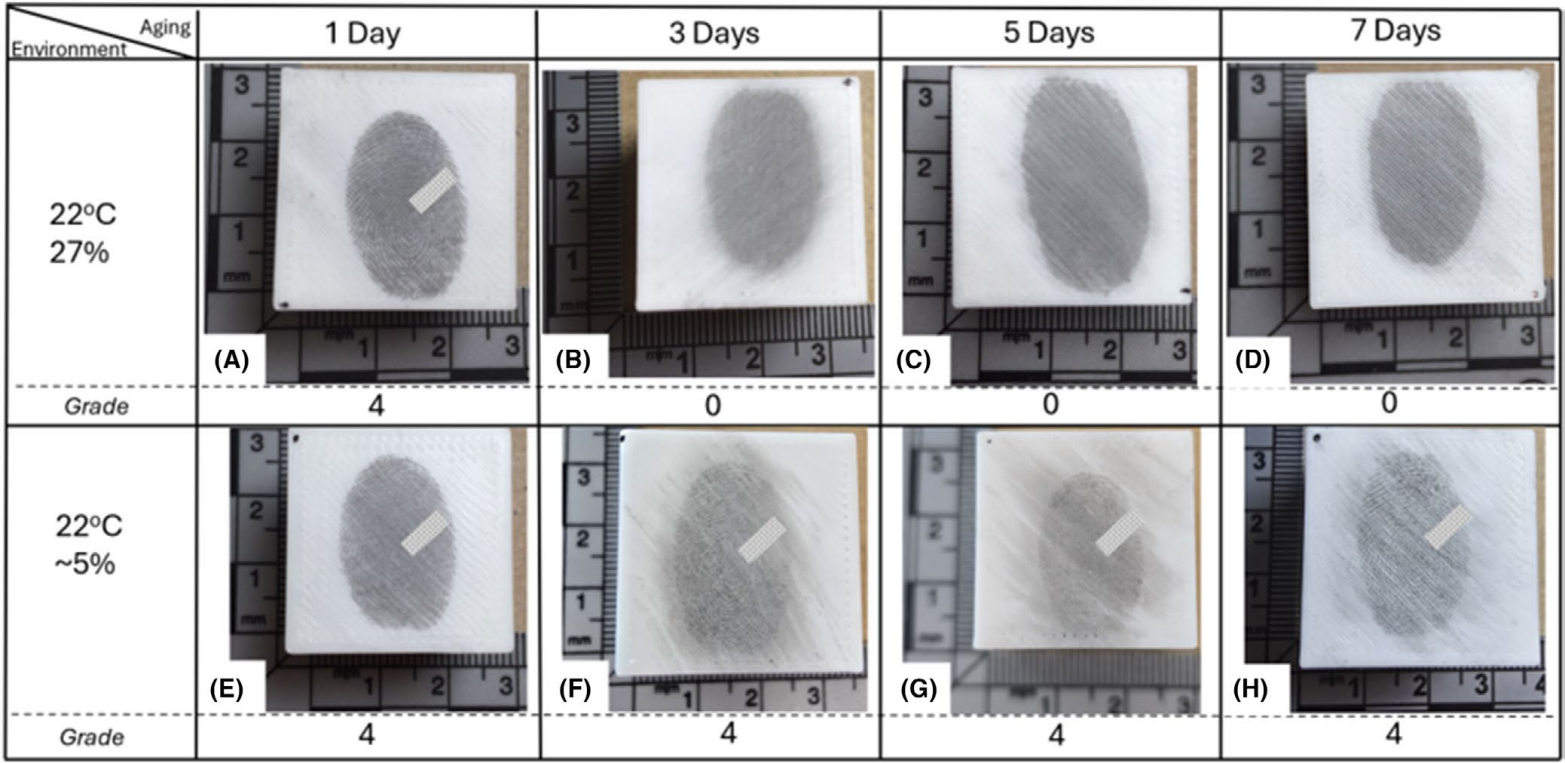
Effects of aging in two different environments over a 7‐day period. Images (A–D) show aging under conditions of 22°C and 27% humidity, while images (E–H) show aging under conditions of 22°C with ~5% humidity.

Latent fingermarks deposited on 3D‐printed surfaces printed using PLA show degradation over time. Figure 5 shows the results of fingermarks deposited and aged over one week in two distinct environments. Over time, fingermarks in environment 1 exhibited gradual degradation, with ridge detail appearing to converge. In contrast, fingermarks aged in environment 2 retained distinct ridge detail throughout the aging period.

The differences observed between the two environments can be attributed to the properties of PLA, a semicrystalline aliphatic polyester with moderate surface energy [24]. This material is also hygroscopic, meaning that it readily absorbs moisture from the surrounding environment [25]. PLA is neither inherently oleophobic nor oleophilic; its moderate surface energy allows for limited interaction with the oils present in latent fingermarks, meaning that fingerprint residues are not strongly repelled but are also not firmly anchored to the surface. High humidity environments are known to result in lower‐quality fingermarks, as elevated moisture can promote extensive background development that obscures ridge details [26]. While this is the commonly accepted explanation for ridge detail loss, it is also possible that the moderate adhesion on PLA accelerates the redistribution or softening of ridge detail over time.

**FIGURE 6:**
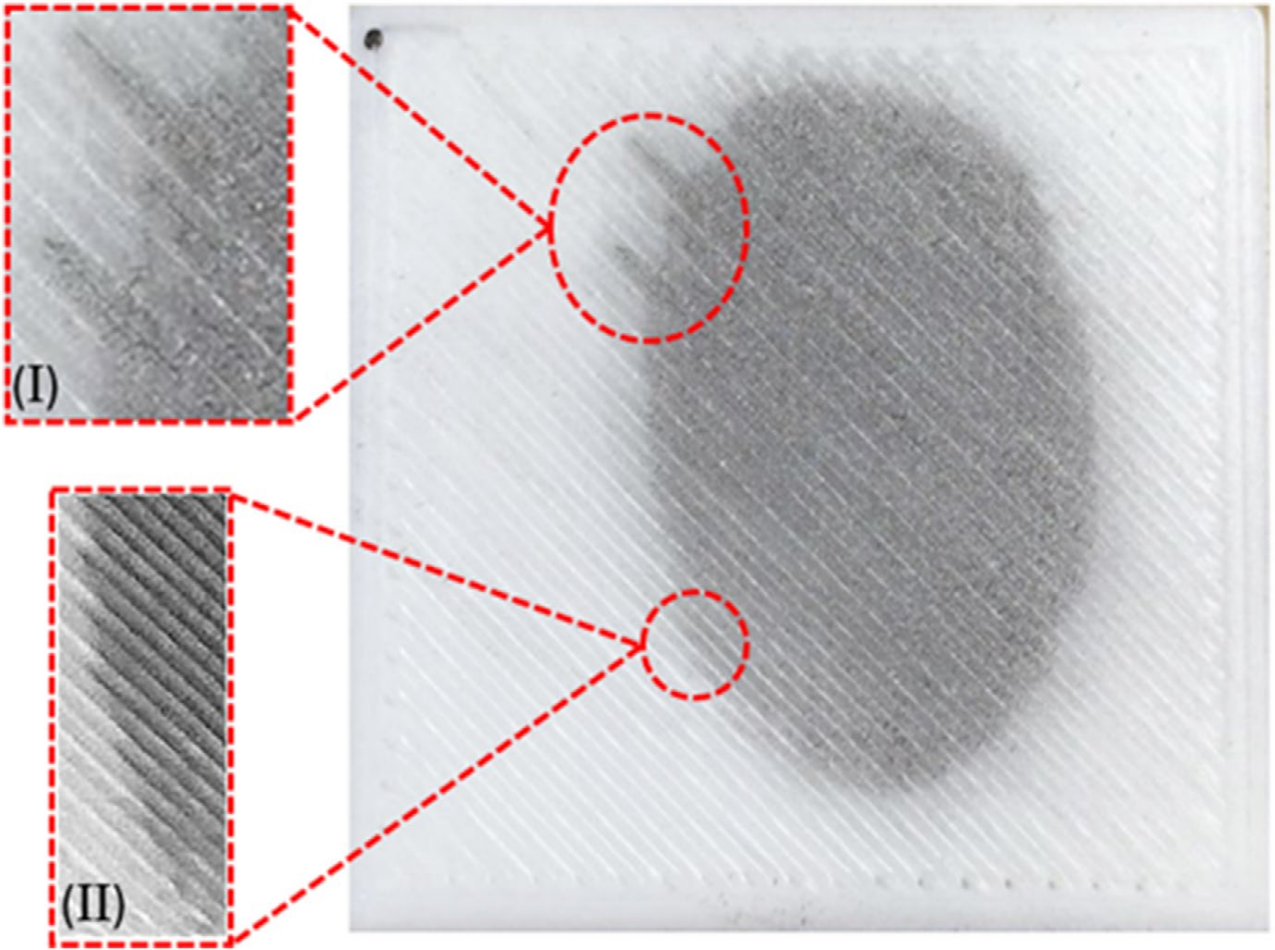
Effect of moisture absorption and the movement of latent fingermark components through the raster lines of PLA where (I ‐II) show amplification of these areas.

In environment 1, the presence of humidity aids moisture absorption by the PLA, altering the stability of fingermark residues, softening ridge structures and leading to the gradual merging of ridge detail. The spread of ridge detail occurs through each raster of the PLA, as shown in Figure 6. This effect is further evident in the non‐uniform appearance of the jagged edges of the fingermark border, as illustrated in Figure 6I,II, where the ridges show irregularities and lack consistency in their alignment across different raster lines. This suggests that moisture absorption by the PLA surface disrupts the integrity of the fingermark, causing a loss of clarity and hindering the overall visualization of the latent fingermark.

In contrast, this effect was not observed in environment 2, where the low‐humidity conditions within the vacuum desiccator minimized water uptake by the PLA. With reduced humidity, fingermark residues remained more stable, preventing ridge detail diffusion and maintaining sharper, more distinct ridge features over the aging period. These findings suggest that the absence of humidity played a critical role in preserving the clarity of friction ridge features. The comparison between environments highlights the significant influence of humidity on the stability and longevity of latent fingermarks on objects printed using PLA.

## 4 CONCLUSION

This research highlights the significant influence of surface texture on the effectiveness of latent fingermark development techniques on 3D‐printed PLA objects. The three distinct surface textures resulting from different printing orientations play a critical role in determining the suitability of various development methods. While powder developers facilitate the recovery of latent fingermarks, the presence of raster lines from the printing process can obscure ridge characteristics, potentially complicating coincident ridge determination. In contrast, CEF proves to be a more effective technique, as its outward development minimizes the interference of raster lines, allowing for clearer ridge detail visualization. However, the material properties of PLA, particularly its hygroscopic nature and moderate surface energy, contribute to the degradation of latent fingermarks over a short period of time under ambient conditions. This results in the diffusion of fingermark components, leading to the loss of ridge detail within a week. Storing the 3D‐printed object under low humidity conditions mitigates this degradation, preserving the integrity of the fingermarks for development and subsequent recovery.

## CONFLICT OF INTEREST STATEMENT

The authors have no competing interests to declare.

## Data Availability

Research data are not shared.
